# Supplementary material for: Modeling glioblastoma heterogeneity as a dynamic network of cell states
Source: Mol Syst Biol. 2021 Sep 16;17(9):e10105. doi: 10.15252/msb.202010105 (PMC8444284; doi:10.15252/msb.202010105)
Supplement: Supplementary file 5 — Source Data for Figure 3 [file MSB-17-e10105-s001.zip › Figure3A_sourcedata/GSEA_3065/nosigdb_state5.GseaPreranked.1622718435914/ALLEN_116_OLIGOL4_6OPALIN.html]

Details for gene set ALLEN\_116\_OLIGOL4\_6OPALIN[GSEA]

|  || Dataset | state5 |
| Phenotype | NoPhenotypeAvailable |
| Upregulated in class | na\_pos |
| GeneSet | ALLEN\_116\_OLIGOL4\_6OPALIN |
| Enrichment Score (ES) | 0.5987822 |
| Normalized Enrichment Score (NES) | 1.7548302 |
| Nominal p-value | 0.010928961 |
| FDR q-value | 0.012685321 |
| FWER p-Value | 0.113 |
Table: GSEA Results Summary

  

Fig 1: Enrichment plot: ALLEN\_116\_OLIGOL4\_6OPALIN      
 Profile of the Running ES Score & Positions of GeneSet Members on the Rank Ordered List

  

| PROBE | GENE SYMBOL | GENE\_TITLE | RANK IN GENE LIST | RANK METRIC SCORE | RUNNING ES | CORE ENRICHMENT || 1 | SCD |  |  | 284 | 0.391 | 0.0798 | Yes |
| 2 | CREB5 |  |  | 321 | 0.373 | 0.1821 | Yes |
| 3 | TMEM63A |  |  | 683 | 0.267 | 0.2180 | Yes |
| 4 | SPP1 |  |  | 724 | 0.256 | 0.2866 | Yes |
| 5 | DOCK10 |  |  | 751 | 0.250 | 0.3550 | Yes |
| 6 | GPRC5B |  |  | 838 | 0.234 | 0.4121 | Yes |
| 7 | PTPRK |  |  | 887 | 0.228 | 0.4716 | Yes |
| 8 | PHLDB1 |  |  | 906 | 0.224 | 0.5335 | Yes |
| 9 | PLP1 |  |  | 1022 | 0.208 | 0.5800 | Yes |
| 10 | RNF220 |  |  | 1713 | 0.132 | 0.5408 | Yes |
| 11 | NCKAP5 |  |  | 1790 | 0.127 | 0.5686 | Yes |
| 12 | CERCAM |  |  | 1837 | 0.124 | 0.5988 | Yes |
| 13 | FRMD4B |  |  | 2516 | 0.078 | 0.5454 | No |
| 14 | FGFR2 |  |  | 2817 | 0.062 | 0.5296 | No |
| 15 | GAB1 |  |  | 3372 | 0.033 | 0.4771 | No |
| 16 | SHROOM4 |  |  | 3578 | 0.024 | 0.4611 | No |
| 17 | CA2 |  |  | 5139 | -0.038 | 0.2979 | No |
| 18 | FBXL7 |  |  | 5313 | -0.045 | 0.2914 | No |
| 19 | C10orf90 |  |  | 5553 | -0.055 | 0.2804 | No |
| 20 | CRYAB |  |  | 8315 | -0.358 | 0.0746 | No |
Table: GSEA details [plain text format]

  

Fig 2: ALLEN\_116\_OLIGOL4\_6OPALIN: Random ES distribution      
 Gene set null distribution of ES for **ALLEN\_116\_OLIGOL4\_6OPALIN**
